# Flexible Sheet-Type Sensor for Noninvasive Measurement of Cellular Oxygen Metabolism on a Culture Dish

**DOI:** 10.1371/journal.pone.0143774

**Published:** 2015-12-01

**Authors:** Mari Kojima, Hiroaki Takehara, Takanori Akagi, Hirofumi Shiono, Takanori Ichiki

**Affiliations:** 1 Department of Bioengineering, School of Engineering, The University of Tokyo, 2-11-16, Yayoi, Bunkyo-ku, Tokyo, Japan; 2 Nikon Corporation, 2-15-3, Konan, Minato-ku, Tokyo, Japan; The Ohio State University, UNITED STATES

## Abstract

A novel flexible sensor was developed for the noninvasive oxygen metabolism measurement of cultivated cells and tissues. This device is composed of a transparent double-layered polymer sheet of ethylene-vinyl alcohol (EVOH) and poly(dimethylsiloxane) (PDMS) having an array of microhole structures of 90 μm diameter and 50 μm depth on its surface. All the microhole structures were equipped with a 1-μm-thick optical chemical sensing layer of platinum porphyrin-fluoropolymer on their bottom. The three-dimensional microstructures of the sensor were fabricated by a newly developed simple and low-cost production method named self-aligned hot embossing. The device was designed to be attached slightly above the cells cultivated on a dish to form a temporarily closed microspace over the target cells during measurement. Since the change in oxygen concentration is relatively fast in the microcompartmentalized culture medium, a rapid evaluation of the oxygen consumption rate is possible by measuring the phosphorescence lifetime of the platinum porphyrin-fluoropolymer. The combined use of the device and an automated optical measurement system enabled the high-throughput sensing of cellular oxygen consumption (100 points/min). We monitored the oxygen metabolism of the human breast cancer cell line MCF7 on a Petri dish and evaluated the oxygen consumption rate to be 0.72 ± 0.12 fmol/min/cell. Furthermore, to demonstrate the utility of the developed sensing system, we demonstrated the mapping of the oxygen consumption rate of rat brain slices and succeeded in visualizing a clear difference among the layer structures of the hippocampus, i.e., the cornu ammonis (CA1 and CA3) and dentate gyrus (DG).

## Introduction

Quality control of cultivated cells or tissue constructs is important in order to conduct safe and effective cell therapy and reliable drug screening [1, 2]. Unfortunately, the quality evaluation of cells, those are in the steady state of dynamic equilibrium of complex biological systems is not straightforward, and hence, broad research has been carried out to explore and establish analytical methods for qualifying cells in a noninvasive manner [3–5]. Cellular metabolism is one of the most important measurement targets, which can be evaluated by measuring certain extracellular parameters such as pH, carbon dioxide, oxygen, nitric oxygen, and glucose. The rate of metabolic reaction reflects the physiological activity of cells [6, 7]. In particular, the oxygen consumption rate has a direct correlation with adenosine triphosphate (ATP) production and energy consumption [8], and thus has been focused on as a valuable parameter in the selection of cells for clinical transplantation. Abe and coworkers reported that bovine embryos with higher oxygen consumption during *in vitro* fertilization (IVF) yielded higher pregnancy rates after embryo transfer (ET) [9, 10]. Thus far, for the measurement of the oxygen consumption rate, microwell array chips [11, 12] and patch-coverslips [13] integrated with an oxygen sensor have been reported to measure the metabolic activity of cells.

However, for ease of applicability and widespread application, a system that enables the *in situ* evaluation of cells or tissue constructs cultivated on a Petri dish is strongly desired. Petri dishes are currently used as a standard format for cell culturing, which is expected to continue. Sheet-shaped tissue constructs, such as organotypic culture of tissue slices and three dimensional engineered tissues, are one of the most suitable tissue models for fundamental study and drug screening, due to the ease of cultivation and microscopic observation [14, 15]. Furthermore, versatile cells such as embryonic stem cells and induced pluripotent stem (iPS) cells are of particular interest since they are expected to lead to innovation in drug discovery and medical treatment in the near future [16]. Combined use of iPS cell technology and sheet-shaped tissue constructs has the potential to treat human diseases [17].

With the above background, this study focuses on the development of the system for the *in situ* measurement of cellular oxygen consumption rate of cells or sheet-shaped tissue constructs cultured in an open culture system. In general, there are two approaches to detect a small change in dissolved oxygen concentration expected in the measurement of cellular oxygen consumption [18]: the use of electrochemical sensors such as Clark-type oxygen sensors and optical chemical sensors using oxygen-sensitive dyes. Electrochemical sensors have the advantages of high-speed response and sensitivity, but are not suitable for measuring changes in concentration in a small space because the sensor itself consumes oxygen during measurement. On the other hand, optical chemical sensors do not consume oxygen during measurement and are relatively suitable for miniaturization. Moreover, they can be used in combination with a microscopic observation system. Therefore, in this study, we decided to integrate optical chemical sensors into our device.

In this study, we have developed a flexible sheet-type sensor that has good compatibility with cells or sheet-shaped tissue constructs cultured on Petri dishes. This device was designed to realize the noninvasive metabolism measurement and optical observation of cells simultaneously. As reported in this paper, prior to cellular measurement, we examined the fundamental characteristics of the device, namely, sensitivity calibration, sealing performance, and cell viability. Cellular measurements were conducted by placing the device over MCF7 breast cancer cells cultivated on Petri dishes. Finally, the oxygen consumption rate mapping of rat brain slices was achieved using the device.

## Material and Methods

### Device design

**Fig 1:**
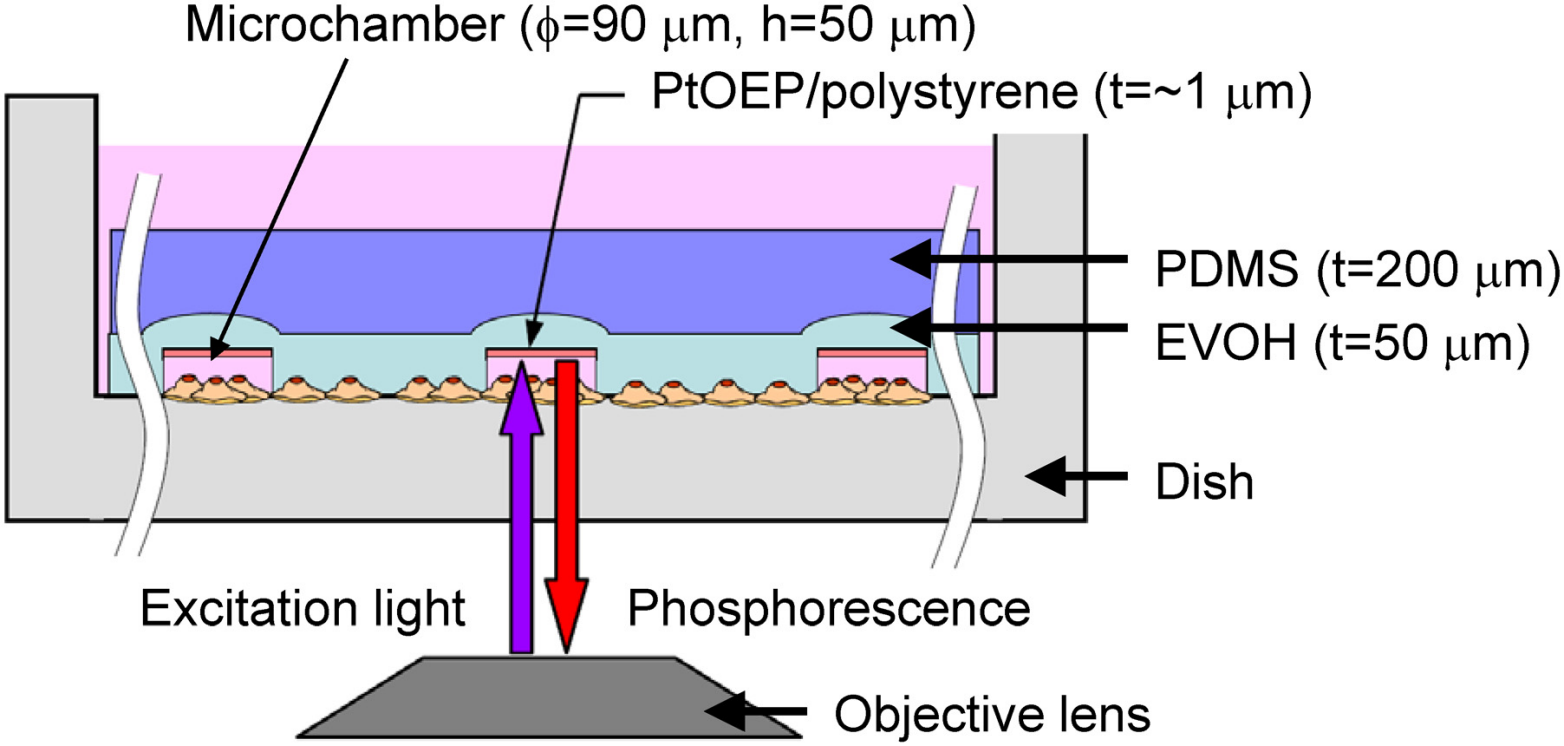
Schematic diagram of flexible sensor sheet. During the measurement, the device is attached to the bottom of the culture dish to form a temporarily closed microspace around the target cells, hence enabling the short-time evaluation of oxygen consumption rate. The device comprises a transparent EVOH/PDMS sheet and an array of microchamber structures (φ 90 μm, 50 μm depth) that contain a 1-μm-thick sensing layer at their bottom.

A schematic diagram of the flexible sensor device is shown in Fig 1. The main body of the device comprises a double-layered sheet of poly(dimethylsiloxane) (PDMS) and 50-μm-thick ethylene-vinyl alcohol (EVOH). Since these polymer materials are soft and transparent in the visible light region, they are suitable for optical imaging and attachment over the cells cultivated in a Petri dish. The EVOH film, which has a very low oxygen permeation rate of 2.964×10^−17^ cm^3^ (STP)cm/cm^2^·s·Pa [19], was used to ensure a tight gas seal during measurement. A circular prototype device with 3 cm diameter was designed; this device had an array of microchamber structures with 90 μm diameter, 50 μm depth, and 200 or 300 μm pitch formed on the surface of the EVOH layer. At the bottom of each microchamber, a 1-μm-thick phosphor sensor layer was formed. Platinum octaethylporphyrin (PtOEP) was used as the phosphor sensor because of its strong phosphorescence with a high quantum yield at room temperature and a relatively long phosphorescence lifetime (~100 μs) [20]. To realize a high-efficiency dissolved oxygen sensor, the probe dye was dispersed in polystyrene (PS) polymer matrix for forming the oxygen sensing layer. PS polymer is chemically and mechanically stable, and highly permeable to oxygen but not to water, metal ions, oxidants, reductants, and proteins [21].

During the measurement, the device is attached to the bottom of a Petri dish to form a temporarily closed microspace around the target cells. The cells directly below the microwell consume oxygen in the microspace. A change in oxygen concentration in the temporary microspace in the vicinity of the cells can be evaluated by measuring the phosphorescence lifetime. The device has three structural features: (1) a transparent flexible polymer sheet, (2) an array of microwell-structured microchambers for the fast and high-sensitivity evaluation of oxygen consumption rate, and (3) a phosphor sensor for the optical measurement of cellular oxygen consumption. The oxygen sensor layer consists of an oxygen-sensitive phosphor sensor mixed into the PS. In the presence of oxygen, the phosphorescence lifetime is shortened as a result of quenching by the collision of oxygen molecules with the phosphor, which deactivates the excited triplet state. The phosphorescence lifetime and dissolved oxygen concentration have a linear relationship in accordance with the Stern-Volmer equation [22].

$$\frac{\tau_{0}}{\tau }=1+K_{SV}\left[O_{2}\right]$$ (1)

Here, τ_0_ is the unquenched emission lifetime, τ is the emission lifetime in the presence of different oxygen concentration, *K*_*sv*_ is the Stern-Volmer constant, and [*O*_*2*_] is the dissolved-oxygen concentration.

### Device fabrication

**Fig 2:**
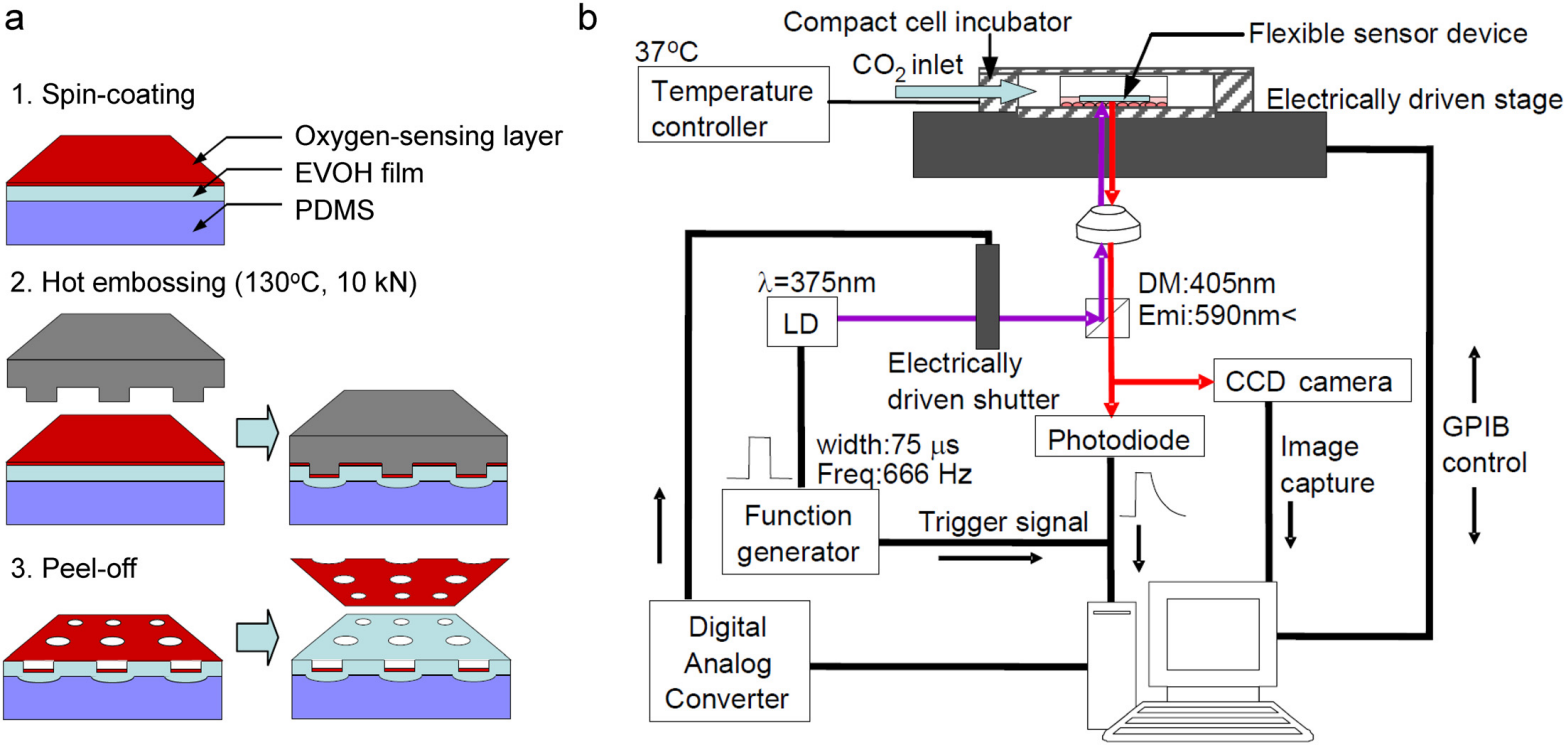
(a) Self-aligned hot embossing method used to fabricate the device. First, a 50-μm-thick EVOH film was laminated onto a PDMS sheet, which was followed by the spin-coating of PtOEP/polystyrene. Subsequently, a silicon micromold was embossed onto the layered polymer under optimized conditions, typically a temperature of 130°C and a force of 10 kN, to form microhole structures containing a self-aligned phosphor sensor layer at their bottom. Finally, the residual phosphor layer outside the hole features was peeled off to obtain the sensor sheet. (b) Experimental setup for *in situ* measurement of cell oxygen consumption. Both phosphorescence lifetime measurement and cultivated cell imaging were automatically performed. The data acquisition speed of the automated sequential measurement system was 100 points/min.

We developed an original microfabrication method, self-aligned hot embossing, that is amenable to the mass production of our device, as shown in Fig 2a. In this process, complex three-dimensional microstructures are easily formed in a single step by hot embossing on a polymer sheet with a multilayered structure made of materials with different mechanical properties.

A 50-μm-thick EVOH film, Soarnol A, was provided by Nippon Synthetic Chemical Industry Co. Japan. A PDMS sheet was formed from Silpot 184 W/C Dow Corning by curing at 100°C for 1000 min with 15% catalyst. The phosphor sensor material was formed by solving 30 wt% PS (average MW 250,000, Acros Organics, USA) and PtOEP dye (purity 98%, Sigma Aldrich, USA) in toluene dehydrate (Wako Pure Chemical Industries, Japan). The concentration of PtOEP in PS was 3.0×10^−5^ mol/dm^3^. A triple-layered sheet of PS-PtOEP/EVOH/PDMS was formed by spin-coating a PtOEP-PS mixture solution on a prelaminated sheet of EVOH/PDMS. Spin-coating was carried out at 1000 rpm for 60 s, and then the toluene solvent was evaporated at 70°C for 90 min.

Subsequently, the triple-layered sheet was hot-embossed with a silicon micromold under optimized conditions, typically a temperature of 130°C and a force of 10 kN, using a nanoimprint machine (NanoimPro Type 210, Nanonics, Japan) to form microhole structures containing self-aligned phosphor sensor layers at their bottom. Since the softening temperatures of EVOH and PS are approximately 160°C and 80–100°C, respectively, only the PS-PtOEP top layer was sheared off at the hole edge by the hot embossing at 130°C. In addition, the use of PDMS elastomer as a back-supporting layer of the EVOH film enables high-fidelity pattern transfer into the EVOH layer from the micromold owing to the sufficient deformability at its back interface. The silicon micromold used here was fabricated by the Bosch etching technique [23]. Finally, the residual phosphor layer remaining on the top surface outside the hole features was peeled off to obtain the flexible sheet-type sensor device.

### Automated optical measurement system

To conduct the high-throughput sensing of cellular oxygen consumption using the developed sheet device, we developed an automatic optical measurement system that enables the phosphorescence lifetime measurement and image acquisition of cultivated cells simultaneously at multiple preprogrammed points.

Fig 2b shows the experimental setup for the oxygen consumption measurement of cells cultivated on a dish using this system. An inverted microscope (Eclipse Ti-U, Nikon, Japan) was used as the platform for optical sensing, i.e., phosphorescence measurement and microscopy imaging. The temperature and CO_2_ concentration in the cultivation dish were maintained using a microscope incubation system (INUBSF-ONICS, Tokai Hit Co., Ltd., Japan) mounted on a microscope stage. A 375 nm UV laser diode (LD) (NDU7212E, Nichia Corp., Japan) driven by an LD driver (ALP-7033CB, Asahi data systems, Japan) was used to excite the oxygen sensor layer. The pulsed operation was trigger-controlled using a function/arbitrary waveform generator (3320A, Agilent, USA). A 590 nm long-pass filter was used to remove any scattered excitation signal from the data collection, and the measurement of phosphorescence lifetime was limited to phosphorescence from inside the microchamber using a pinhole. A charge-coupled device (CCD) color camera (CS5270B, Toshiba Teli Co., Japan) connected to the base port of the microscope was used to obtain microscopy images. The phosphorescent emission light was converted to an electric signal through an avalanche photodiode (APD) module (C5460, Hamamatsu, Japan). Data were sent to a computer digitizer/oscilloscope (PCI-5114, National Instruments, USA). Phosphorescence lifetime was calculated by fitting an exponential function to the decay curve. The sampling rate was 125 Hz, the recording time was 450 μs, and the number of repetitions was 185. Cellular image data were obtained using a high-quality monochrome image acquisition device (IMAQ PCI-1409, National Instruments, USA) via a digital-to-analog converter (USB-6008, National Instruments, Austin, TX, USA).

Excitation light was pulsed for a short time at a low energy to avoid cytotoxicity (LD energy 0.375 kJ/m^2^, irradiation time 75 μs, repetition time 1.5 ms). It is known that there is no effect on cell viability when 99 kJ/m^2^ UVA is irradiated to the breast cancer cell line MCF7 for 40 min [24].

LabVIEW software (LabVIEW 2011, National Instruments, USA) was used to control the system for driving an electric shutter (VMM-T1, Uniblitz, USA) via a digital-to-analog converter (USB-6008, National Instruments, Austin, TX, USA) and a motor-driven x-y stage (FC-101G, Sigma Koki, Japan) and for analyzing data.

By the step-and-repeat optical measurement of each microhole using the automated system, the oxygen metabolism on a cultivated dish could be evaluated at multiple points. The data acquisition speed achieved in this study was 100 points/min.

### Oxygen sensor calibration

Prior to cellular measurements, the calibration of the phosphorescence lifetime versus oxygen concentration was conducted. The flexible sensor device was submerged inside MCF7 culture medium RPMI solution (Roswell Park Memorial Institute 1640, Gibco, USA) and the sensor output was quantified by determining the average phosphorescence lifetime and the standard deviation for four or eight sensors in the array under various dissolved oxygen concentrations, where dissolved oxygen concentration was decreased by N_2_ bubbling. RPMI solution with 0 mg/L dissolved oxygen was obtained by adding sodium sulfite (Wako Pure Chemical Industries, Japan). A digital dissolved oxygen concentration sensor (DO-5509-A, Fuso, Japan) was used to prepare the standard solution used in the calibration experiment.

### Cellular oxygen consumption measurement of MCF7

The human breast cancer cell line MCF7 was cultured at 37°C in 5% CO_2_ and 95% air in RPMI 1640 medium supplemented with 10% fetal calf serum, 10 mg/L kanamycin sulfate, and 10,000 unit/ml penicillin and streptomycin (Gibco, USA). Cells were cultured to confluency in 30 mm glass-based dishes (Iwaki, Asahi Techno Glass Co., Tokyo, Japan). During the cellular oxygen consumption measurement, the cultivation dishes were set in a compact cell incubator mounted on a microscope and the flexible sensor devices were attached onto the dish with the pressure 10 kPa.

### Oxygen consumption rate mapping of the acute brain slice of rat

All experimental protocols were approved by the Animal Experiment Committee of the School of Medicine, The University of Tokyo (M-P10-110). Acute brain slices of a rat were prepared using the protocol described by Fuller *et al*. [25]. A16-day-old rat pup (Japan SLC, Inc., Shizuoka, Japan) anesthetized with 2–4% isoflurane inhalation was decapitated and the hippocampus was rapidly removed and immersed in 37°C artificial cerebrospinal fluid (ACSF) medium. The brain was cut transversely through the hippocampus using a tissue chopper at intervals of 350 μm. The ACSF medium was composed of 125 mM NaCl, 1.25 mM NaH_2_PO_4_, 2.5 mM KCl, 26 mM NaHCO_3_, 2 mM CaCl_2_, 1 mM MgCl_2_, 20 mM D-glucose (all obtained from Wako Pure Chemical Industries, Japan). The ACSF medium was saturated with 90% O_2_/10% CO_2_ for 10 min and its osmolarity was adjusted in the range of 310–315 mOsm/kg. The cultivation dish with the flexible sensor device and a brain slice submerged in ACSF medium was set in a compact cell incubator mounted on a microscope. The brain slice was attached onto a polyvinylidene difluoride membrane (Millipore, Bedford, MA) to ease handling, which was followed by oxygen metabolism measurement using the device. The oxygen consumption rates were analyzed for 4×4 microchambers for the oxygen consumption mapping of the brain.

### Statistical analysis

The statistical significance of differences between groups was validated by the analysis of variance (ANOVA) and Tukey’s test. A probability level of p<0.05 was considered to be significant.

## Results and Discussion

### Characterization of the flexible sensor device

**Fig 3:**
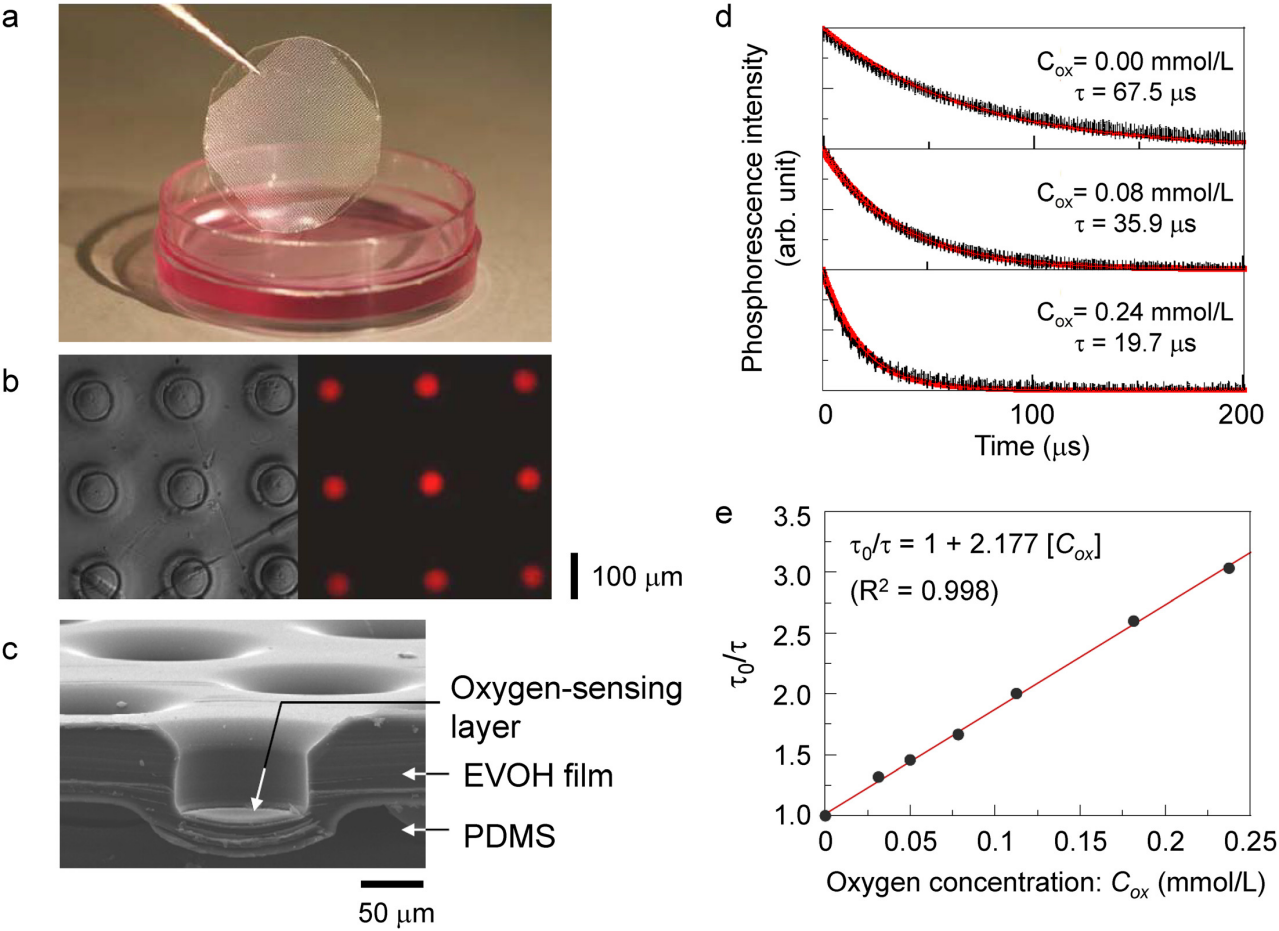
(a) Photograph of the flexible sensor device. The device comprises a transparent EVOH/PDMS sheet and an array of microchamber structures. (b) Bright-field image and phosphorescence image of the sheet. (c) SEM image of the sheet. Each microchamber has a 90 μm diameter and a 50 μm depth, and contains a 1-μm-thick sensing layer at its bottom. (d) Phosphorescence lifetime τ measurement at three different dissolved oxygen concentrations (C_ox_ = 0.00, 0.08, and 0.24 mmol/L). In the presence of oxygen, τdecreased as a result of the quenching of the excited triplet state by the collision of oxygen molecules with phosphor molecules. (e) Oxygen sensor calibration. τ and C_ox_ exhibited a linear relationship in accordance with the Stern-Volmer equation.

Fig 3a–3c show a photograph, a set of bright-field image and phosphorescence image, and a cross-sectional scanning electron microscopy (SEM) image of the microchamber of the flexible sensor device, respectively. An array of microchamber structures integrated with a 1-μm-thick sensing layer at their bottom was fabricated on a transparent flexible polymer sheet. The self-aligned hot embossing technique developed in this study enabled the simple and high-productivity fabrication of the device.

Prior to the measurement of cells, we evaluated the fundamental characteristics of the oxygen sensor. Fig 3d shows the decay curves of the phosphorescence at different dissolved oxygen concentrations. The phosphorescence lifetime decreased with increasing concentration of dissolved oxygen. The lifetimes were 19.73 ± 0.61 μs at 0.24 mmol/L, 35.86 ± 0.55 μs at 0.08 mmol/L, and 67.45 ± 0.11 μs at 0.00 mmol/L. Thus, the sensor has a small standard deviation of less than 3% at high dissolved oxygen concentration condition, and less than 1% at low dissolved oxygen concentration. The variability of the sensor increased with increasing dissolved oxygen concentration, because the signal-to-noise ratio of the light detected by the APD decreased with increasing oxygen concentration to quench the phosphorescent emission.

The calibration plot shown in Fig 3e was obtained by plotting the reciprocal of each standardized phosphorescence lifetime against the dissolved oxygen concentration. The phosphorescence lifetime and dissolved oxygen concentration exhibited a linear relationship in accordance with the Stern-Volmer equation. The calibration plot can be used to convert the phosphorescence lifetime to the dissolved oxygen concentration and to evaluate the cellular oxygen consumption.

### Evaluation of the noninvasiveness of the flexible sensor device

During the cellular oxygen consumption measurement, the culture medium around the target cells is temporarily closed inside the microchamber to measure the change in phosphorescence lifetime, which depends on the oxygen concentration of the culture medium. The optimization of the pressure used to attach the flexible sensor device onto the cells is required to realize compartmentalization without the destruction and/or detachment of the cells. Prior to the cellular measurement, we investigated the sealing performance and cell viability to determine the appropriate pressure.

**Fig 4:**
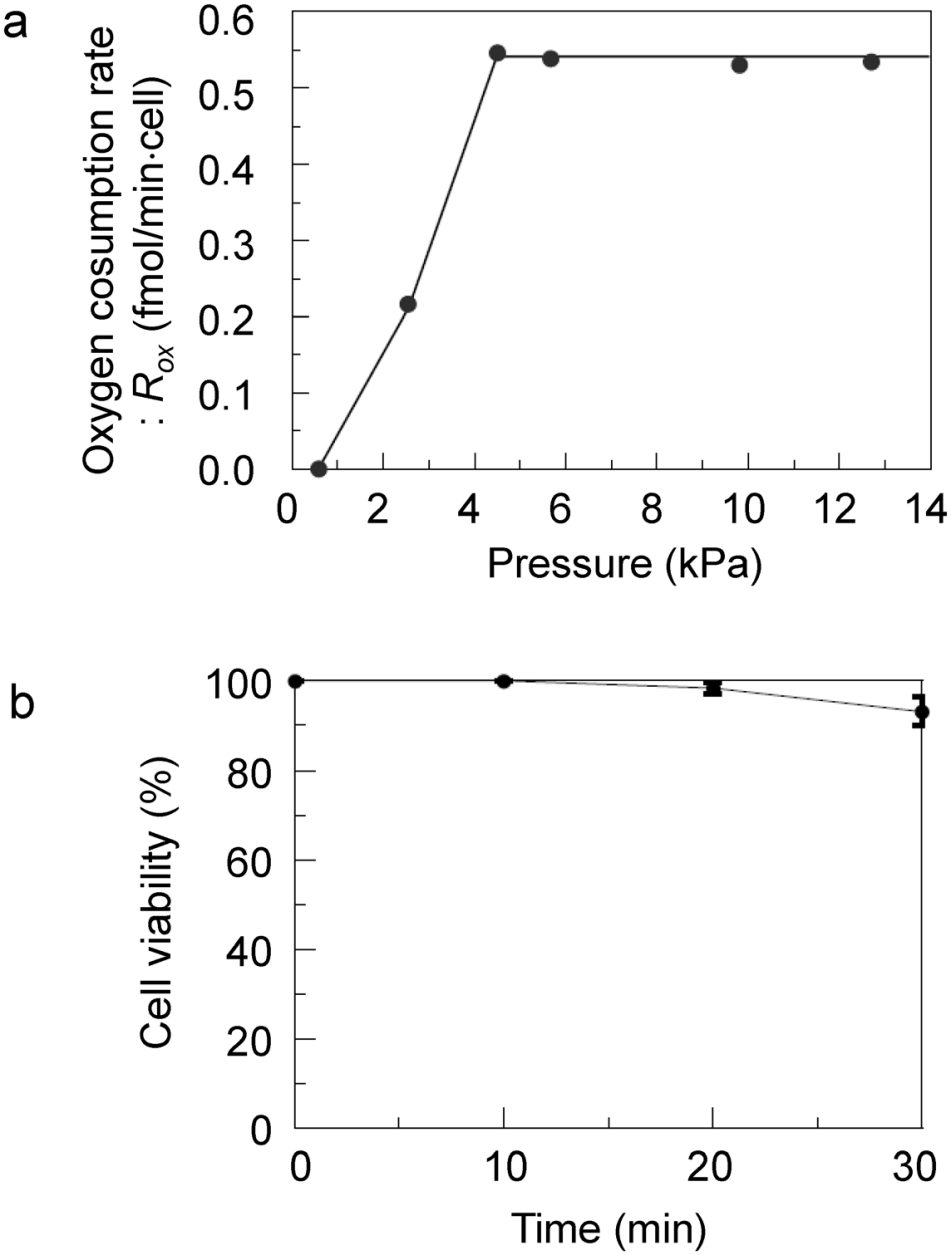
(a) Dependence of oxygen consumption rate (R_ox_) on pressure. R_ox_ converges at pressures of 4.5–12.7 kPa because of the high sealing performance of the microchamber. (b) Results of trypan blue cell viability assay. Each point represents the mean with standard deviation (S.D.) of 3 measurements. The cell viability on 30 min was statistically different from that on 0, 10, 20 min (p<0.05, respectively, the Tukey test).

First, we evaluated the sealing performance of a microchamber under various pressures. The flexible sensor device was placed on the confluent MCF7 cells cultured on a dish, and measurements were carried out under different pressures. The oxygen consumption rate per cell was calculated, and the pressure range in which the oxygen consumption rate converged was determined as the range in which the microchamber was sealed. The pressure range was limited to 0–12.7 kPa because the cells became detached above a pressure of 20 kPa. The average oxygen consumption rate per cell calculated from the change in the dissolved oxygen concentration of the microchamber under various pressures is shown in Fig 4a. The oxygen consumption rate converges at pressures of 4.5–12.7 kPa because of the high sealing performance of the microchamber. It was also anticipated that the long-time attachment of the device would lead to oxygen deficiency because EVOH has an excellent gas barrier property. Therefore, we evaluated the viability of cells outside the microchamber by trypan blue cell viability assay. As shown in Fig 4b, cell viability slightly decreased after 10 min but there was no change before 10 min. Thus, to ensure minimum cell damage, oxygen consumption rate measurement was conducted by attaching the device for less than 10 min at a pressure of 10 kPa.

### Oxygen consumption rate measurement of the human breast cancer cell MCF7

**Fig 5:**
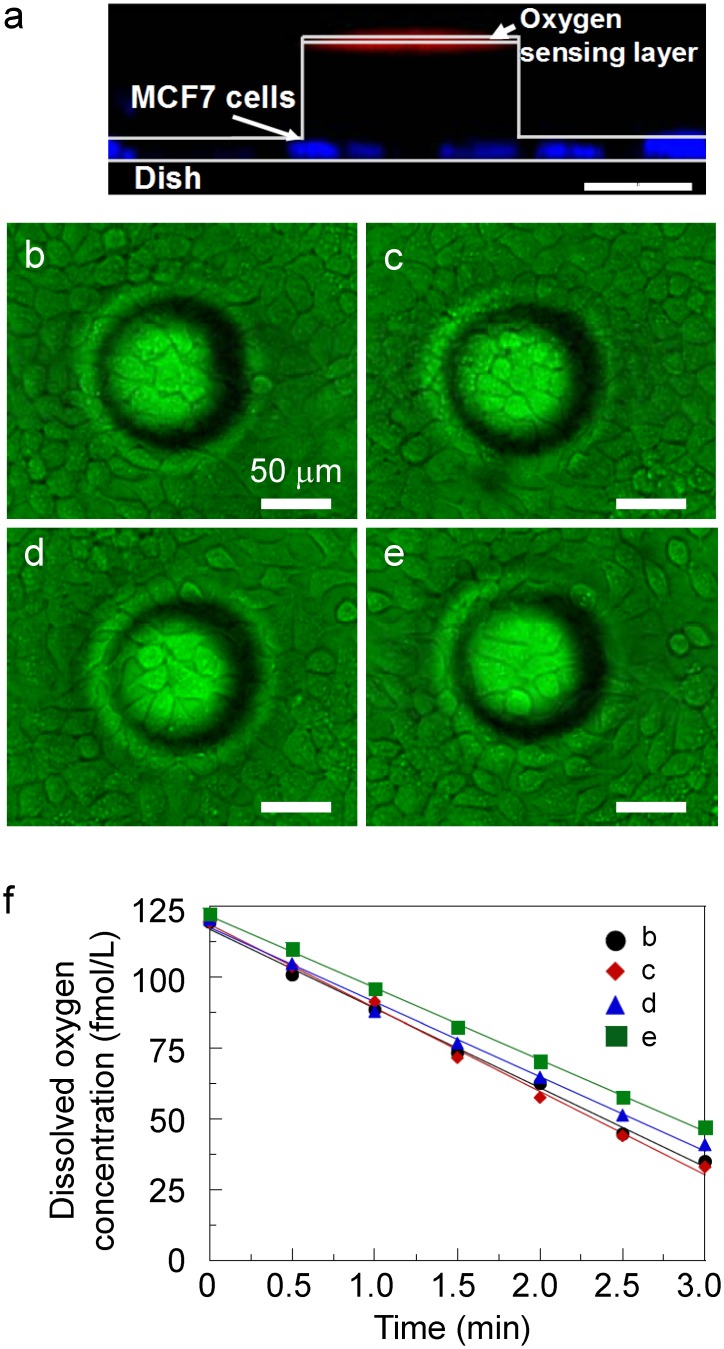
(a) Cross-sectional image of MCF7 and flexible sensor sheet. Scale bar: 50 μm. (b)-(e) Bright-field images of MCF7 and flexible sensor sheet. Scale bar: 50 μm. The numbers of cells in the chambers are n = 18, 20, 15, and 13, respectively. (f) R_ox_ measurement performed using the device. The linear decrease in oxygen concentration was measured as a result of cell respiration.

To evaluate the attachment of the flexible sensor device during measurement of the metabolism of the MCF7, cross-sectional imaging was carried out using a confocal microscope and the obtained image is shown in Fig 5a. A red fluorescent sensor layer was observed above blue fluorescent MCF7 cells. The MCF7 cells were DNA-stained by Hoechst 33342. This image shows that the measurement environment comprising microchamber structures formed a sealed space around the cells. Then, cellular measurement was conducted by attaching the device to MCF7 breast cancer cells cultivated on Petri dishes as shown in Fig 5b–5e. The linear decrease in oxygen concentration was measured as a result of cell respiration. This linear decrease indicated that the metabolism of cells was not affected by the sensor nor illumination. Furthermore, as shown in Fig 5f, an increase in the number of cells inside the microchamber structures including tapered area led to a rapid decrease in oxygen consumption, which means that the oxygen consumption increased with the number of cells. By dividing the slope of the dissolved oxygen concentration by the number of cells, the oxygen consumption rate of MCF7 was evaluated to be 0.72 ± 0.12 fmol/min/cell (N = 22), which was in agreement with previously reported values [11]. This suggests that the flexible sensor device can be used to evaluate the oxygen metabolism of cells cultured on a Petri dish.

### Oxygen consumption rate mapping of acute brain slices of rat

**Fig 6:**
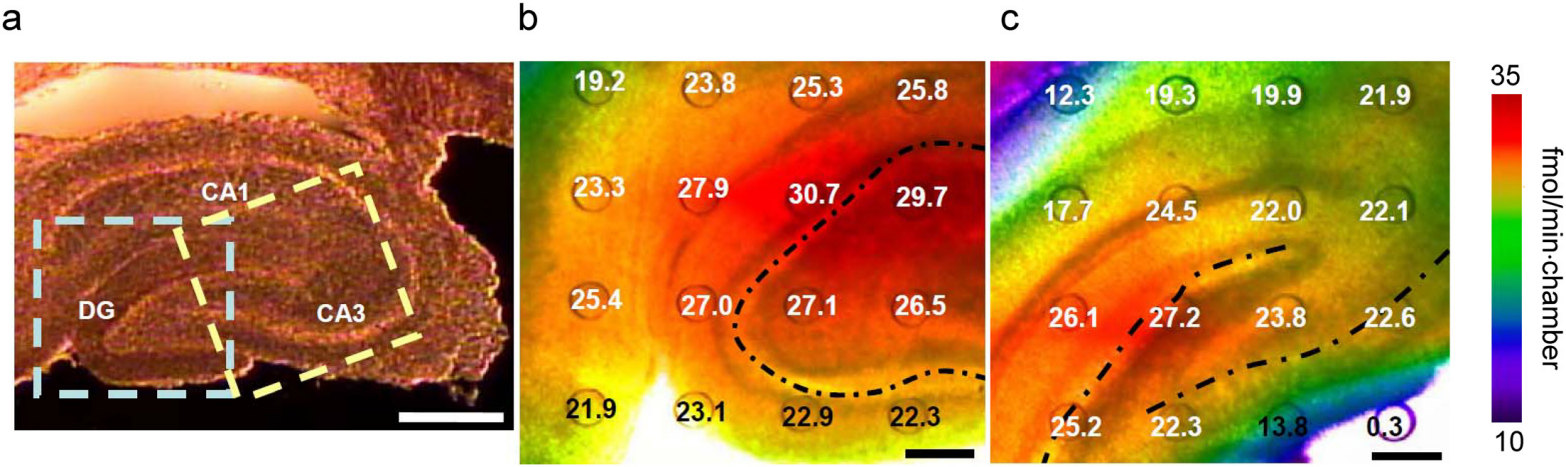
(a) Bright-field image of rat hippocampus slice. Scale bar: 500 μm. (b) Oxygen concentration rate mapping of rat hippocampus slice indicated with blue box in a. (c) Oxygen concentration rate mapping indicated with yellow box in a. CA1 and CA3 are pyramidal cells of the hippocampus and DG is the dentate gyrus. Broken lines show neuronal cell bodies. This result indicates that the oxygen consumption rate of DG is higher than those of CA3 and CA1, and the oxygen consumption rate around neuronal cell bodies is higher than in the molecular layers. Scale bar: 200 μm.

The oxygen consumption rate mapping (4×4) of rat brain slices was performed. The flexible sensor device was attached to the acute brain slices of a rat during the measurement, and the change in dissolved oxygen concentration was measured using the automatic optical system. Fig 6a–6c show the results of the oxygen consumption rate mapping of a rat hippocampus slice. It is known that the oxygen consumption rate of the brain is 10 times higher than that of other tissues, and indeed we observed a change in oxygen concentration in short-time measurement. The oxygen consumption rate around the neuronal cell bodies was 25–30 fmol/min, compared with 10–25 fmol/min in the molecular layer that is a relatively cell-free layer. Furthermore, as shown in Fig 6b and 6c, differences in oxygen consumption rate among the brain layers such as the cornu ammonis (CA1 and CA3) and dentate gyrus (DG) were observed. Thus, the flexible sensor device enabled the real-time mapping of the metabolic activity of cultivated tissues, and is expected to be widely used in the fields of drug development and neuroscience research.

## Conclusions

We have developed a novel flexible sensor for the *in situ* and spatiotemporal monitoring of the oxygen consumption of cultivated cells and tissues with fmol/min resolution. This device provides a practical means for the *in situ* evaluation of cells cultivated on the flat surface of a Petri dish. It comprised a transparent sheet and an array of microhole structures with an oxygen sensing layer at their bottom, and was fabricated by a self-aligned hot embossing method, which was newly developed in this study to enable the low-cost and high-productivity formation of the three-dimensional structured sensor. Moreover, it showed an accuracy which is less than 1% at low dissolved oxygen concentrations. We also developed an automatic optical measurement system for the simultaneous phosphorescence lifetime measurement (100 points/min) and imaging of cultivated cells.

To demonstrate the operation of our flexible sensor, we measured the oxygen consumption of MCF7 cells and rat’s acute brain slices of a rat. The oxygen consumption rate of MCF7 was evaluated to be 0.72 ± 0.12 fmol/min/cell, which was in agreement with previously reported values. The oxygen consumption rate mapping of the brain slices showed a difference in oxygen consumption rate among the brain layers. Thus, by the combined use of the device and the automatic optical measurement system, we successfully measured the oxygen consumption rate of local groups of cells cultivated on a dish and carried out the real-time mapping of the metabolic activity of cultivated tissues. The present technology is expected to be further extended to include other types of sensing extracellular parameters such as pH, CO_2_, and NO by incorporating different phosphor materials as the sensing layer. This system has widespread applications for drug toxicity testing and for evaluating culture parameters during the development of cell processing technology.
